# Comparison of Chloroplast Genome Sequences of *Saxifraga umbellulata* var. *pectinata* in Qinghai–Xizang Plateau

**DOI:** 10.3390/genes16070789

**Published:** 2025-06-30

**Authors:** Cui Wang, Kaidi Su, Qiwen Li, Rui Sun, Haoyu Liu, Jingxuan Du, Jinping Li, Likuan Liu

**Affiliations:** 1 Qinghai Provincial Key Laboratory of Medicinal Plant and Animal Resources of Qinghai-Tibet Plateau, School of Life Sciences, Qinghai Normal University, Xining 810008, China; 13299879387@163.com (C.W.); 15169936717@163.com (K.S.); liqiwen1205@163.com (Q.L.); 13700360291@163.com (R.S.); 15003655796@163.com (H.L.); dujingxuan0303@163.com (J.D.); 2 Academy of Plateau Science Sustainability, Xining 810008, China

**Keywords:** *Saxifraga*, chloroplast genome, genome comparison, molecular evolution, phylogeny, Tibetan Plateau

## Abstract

Background: *Saxifraga umbellulata* var. *pectinata* (Saxifragaceae) is recognized as a genuine medicinal material from the Qinghai–Tibet Plateau in China. This paper presents the chloroplast (cp) genome of *S. umbellulata* var. *pectinata*, marking the first report for this genus. The Tibetan medicinal plants documented in ‘Chinese Medicinal Plant Resources’ are associated with their chloroplast genomes and medicinal mechanisms. Objective: In order to resolve any potential ambiguity in conventional classifications, this study reconstructs the evolutionary position of *S. umbellulata* var. *pectinata* within the genus by comparing its chloroplast genetic information with that of other groupings. Methods: The chloroplast genome of *S. umbellulata* var. *pectinata* was sequenced using the Illumina NovaSeq 6000 platform. Subsequent sequence assembly, annotation, and characterization were performed using bioinformatics analysis. The NJ phylogenetic tree was constructed using MEGA 7.0 software. Results: The complete chloroplast genome of *S. umbellulata* var. *pectinata* is 146,549 bp in length, comprising four subregions: a large single-copy (LSC) region of 79,318 bp and a small single-copy (SSC) region of 16,390 bp, separated by a pair of inverted repeat (IR) regions each 25,421 bp long. This cp genome contains 131 genes, including 86 protein-coding genes, 37 tRNA genes, and 8 rRNA genes. The overall GC content is 38.1%. Phylogenetic analysis based on 20 cp genomes indicates that *S. umbellulata* var. *pectinata* is closely related to *Saxifraga sinomontana* and *Saxifraga stolonifera*.

## 1. Introduction

*Saxifraga* (Saxifragaceae), the largest and most complex genus within the family, is characterized predominantly by cold-tolerant perennial herbs, while some may be annual or biennial herbs [1]. This genus, belonging to the Saxifragaceae family, exhibits the richest diversity of species and the most complex taxonomy. Furthermore, it constitutes a distinctive component of temperate to polar climatic zones as well as mountain to alpine vegetation zones in the Northern Hemisphere [2]. *Saxifraga* primarily thrives in the northern temperate zone. In China, this genus comprises 7 subgenera, 28 sections, and approximately 500 species, distributed across South China, North China, and most densely in Southwest China. About 220 *Saxifraga* species occur nationwide, with the Himalayas and Hengduan Mountains hosting the highest diversity. The Hengduan Mountains alone harbor 75 species, representing nearly half of the genus’ global total [3,4]. *Saxifraga* serves as a medicinal herb with multiple pharmacological properties, traditionally employed to dispel wind, clear heat, cool blood, and detoxify. It also demonstrates anti-inflammatory and swelling-reducing effects, showing efficacy in treating eczema, frostbite, traumatic hemorrhage, and cough [5]. The Qinghai–Tibet Plateau is a region in China where Saxifragaceae plants are highly concentrated [6]. Moreover, it possesses significant medicinal value. The *Annals of Traditional Chinese Medicine Resources* in China records 155 medicinal plant species across 24 genera (including subgenera) in this region [7]. Fifty-nine species (including subspecies) of Saxifragaceae are employed in Tibetan medicine, most demonstrating distinctive therapeutic applications characteristic of this tradition. Representative species include *Chrysosplenium nudicaule*, *Chrysosplenium nepalense*, and *Saxifraga stolonifera*, etc. [8]. In East Asia, multiple *Saxifraga* species serve as traditional folk medicines. Of note, *Saxifraga umbellulata* var. *pectinata*—a medicinal plant in Tibet—is widely used by Tibetan communities to treat hepatobiliary disorders [9]. *S. umbellulata* var. *pectinata* is both a medicinal and edible plant with significant economic value, as documented in the *Records of Traditional Chinese Medicine Resources* of China. However, genetic studies on this taxon remain limited globally, particularly concerning its taxonomic status.

Chloroplasts in advanced plant species function as semi-autonomous organelles that possess a relatively conserved genetic system [10]. Serving as the main sites for photosynthesis, these organelles house a fully autonomous genome. The structure of their genes exhibits a significant degree of stability, conservative characteristics, and a gradual pace of evolution—rendering chloroplast genomes important molecular instruments for investigating species evolution, classification, and phylogenetic relationships [11]. The chloroplast genome of higher plants typically comprises a large single-copy (LSC) region, a small single-copy (SSC) region, and two inverted repeat (IR) regions, forming a circular double-stranded DNA structure ranging from 120 to 180 kb [12]. Chen et al. revealed through comparative analysis that *Saxifraga* chloroplast genomes range from 146,549 bp to 151,066 bp, displaying a conserved quadripartite structure with GC content between 37.8% and 38.1%. Notably, high-altitude species exhibit positive selection in ndh1 and ycf1 genes. These genes facilitate electron transport in photosystem I and chloroplast protein trafficking, potentially enabling adaptation to high-altitude stressors such as intense irradiance and low CO_2_ concentrations [13]. This study investigates the structure and function of the chloroplast genome in *S. umbellulata* var. *pectinata*, laying the foundation for subsequent research on genetic diversity and population genetics.

## 2. Materials and Methods

### 2.1. Test Materials

**Figure 1:**
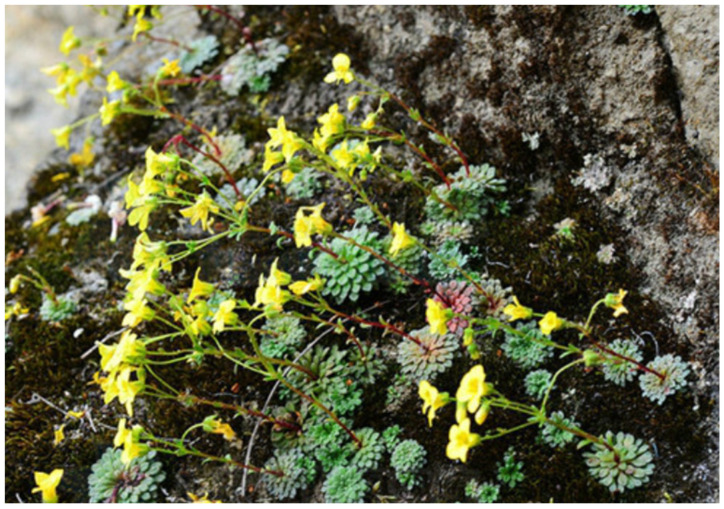
Photo of *S. umbellulata* var. *Pectinata.* Notes: Nook at Lhasa City. Its basal leaf blade margin with cartilaginous teeth and yellow petals, distinguishable from other variants.

Leaves of *S. umbellulata* var. *pectinata* were collected from Duodigou Mountain (waist area), Chengguan District, Lhasa City, Tibet China Autonomous Region (elevation: 4231.61 m; coordinates: 91°11′38″ E, 29°43′29″ N). Specimens were authenticated by Prof. Yang Zeng at the College of Life Sciences, Qinghai Normal University, and deposited in the Herbarium of Zhengzhou University’s College of Life Sciences (Voucher no.: BCHRC2020-0819) (Figure 1).

### 2.2. DNA Extraction and Sequencing

*S. umbellulata* var. *pectinata*, collected from the Tibet Autonomous Region, was preserved in liquid nitrogen (Qinghai Lainer Biotechnology, Xining, China). Through the use of a modified cetyltrimethylammonium bromide (CTAB) procedure [14], whole-genome DNA was isolated from leaves. The Illumina NovaSeq 6000 platform was used for sequencing all DNA sequences. Clean reads provided over 5000 times coverage of each complete cp genome.

### 2.3. Chloroplast Genome Assembly and Annotation

Through base calling analysis, the raw imaging data obtained from sequencing is converted into FASTQ-format raw data [15]. Genome assembly is conducted using SPAdes v3.10.1 software [16]. The results are then compared with the chloroplast genome of a closely related species, *Saxifraga sinomontana* (MN_104589.1), using blastn, filtering for sequences with >90% coverage and >500 bp length, which are retained as candidate sequences. The relationships among the candidate sequences are assessed based on the alignment order of the closely related species to verify their circularity. Finally, gaps in the sequences are filled using Gapcloser v 1.12 to obtain a complete genomic sequence. After submission and annotation through BankIt to the NCBI platform, the sequence is assigned the accession number MZ901175.1.

### 2.4. Analysis of Codon Usage Frequency and Simple Repeat Sequence (SSR) of Chloroplast Genome

Simple repeat sequence analysis (SSR) was performed using MISA v 2.1 [17] (https://webblast.ipk-gatersleben.de/misa/, accessed on 10 March 2025), and the detection parameters (minimum number of repeats) were set to 8, 4, 4, 3, 3, and 3, respectively.

### 2.5. Comparative Analysis of the Whole Chloroplast Genome of Saxifraga

IR boundary analysis was performed on the chloroplast genomes of 18 species of *S. umbellulata* var. *pectinata* and their chloroplast genome sequences were saved in gb format. The online tool IRscope was used for further exhibiting gene distribution at the boundaries of SSC, LSC, IRa, and IRb [18] (https://irscope.shinyapps.io/irapp/, accessed on 3 April 2025). The annotation of chloroplast genome sequences was formatted for conversion, and a visual analysis of the homology of the entire chloroplast genomes of 20 species was conducted using the Shufle-LAGAN mode of the online software mVISTA v2.0 (https://genome.lbl.gov/vista/mvista/submit.shtml, accessed on 3 April 2025). This analysis aimed to observe the differences in chloroplast genomes, with *S. umbellulata* var. *pectinata* (MZ901175.1) selected as the reference sequence [19].

### 2.6. Phylogenetic Analysis

The chloroplast genome of *S. umbellulata* var. *pectinata* was aligned with 19 other chloroplast genomes using MAFFT v7.490 (Katoh sequence and Standley genome sequence (downloaded from GenBank)). Saxifragaceae sequences were downloaded from NCBI: *S. sinomontana* (MN 104589.1), *S. stolonifera* (NC 037882.1), *Chrysosplenium sinicum* (MK 814606.1), *Chrysosplenium lanuginosum* (MK 814607.1), *Chrysosplenium macrophyllum* (MK973001.3), *Chrysosplenium flagelliferum* (MN729584.1), *Micranthes melanocentra* (NC_056191.1), *Bergenia scopulosa* (NC 036061.1), *Rodgersia aesculifolia* (MW327540.1), *Oresitrophe rupifraga* (NC 037514.1), and *Tiarella trifoliata* (NC 042929.1), along with eleven closely related species. *Rheum tanguticum* (NC_046695.1) and *Fallopia aubertii* (MW664925.1) were designated as outgroups. Evolutionary relationships among these 20 *Saxifraga* species were analyzed by constructing a phylogenetic tree based on the aligned chloroplast genomes using the neighbor-joining (NJ) method in MEGA 7.0 with the Tamura–Nei model, supported by 1000 bootstrap replicates.

## 3. Results

### 3.1. Basic Characteristics of Chloroplast Genome of S. umbellulata var. pectinata

**Figure 2:**
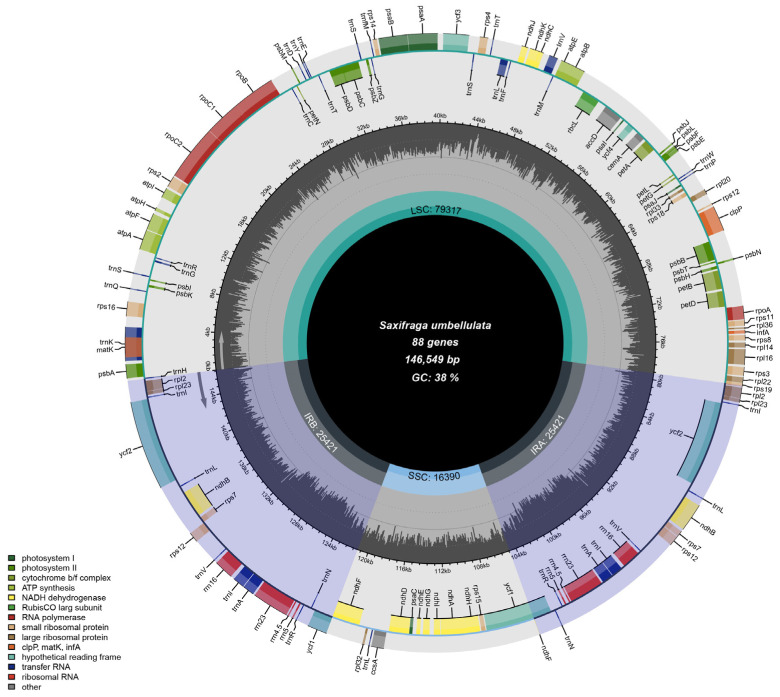
Complete chloroplast genome map of *S. umbellulata* var. *pectinata*. The different colors represent genes in each group.

The chloroplast genome of *S. umbellulata* var. *pectinata*, with a size of 146,549 bp, has been submitted to NCBI (GenBank accession number: MZ901175.1). This chloroplast genome exhibits a typical circular quadripartite structure, comprising four distinct regions: the large single-copy region (LSC), the small single-copy region (SSC), and two inverted repeat regions (IRs). Specifically, the LSC region spans 79,318 bp, the SSC region measures 16,390 bp, and each of the two IR regions is 25,421 bp in length (Figure 2). The chloroplast genome of *S. umbellulata* var. *pectinata* encodes a total of 131 genes, including 86 protein-coding genes, 8 rRNA genes, and 37 tRNA genes (Table 1). The total GC content of the chloroplast genome is 38.1%. Specifically, the GC contents of the large single-copy (LSC), small single-copy (SSC), and inverted repeat (IR) regions are 36.2%, 32.4%, and 42.8%, respectively. Based on their biological functions, the 86 protein-coding genes can be classified into three categories: 44 genes involved in light cooperation, 36 genes associated with expression regulation, and 6 genes with other functions.

### 3.2. Relative Synonymous Codon Usage Frequency (RSCU), Repetitive Sequence, and SSR Analysis of S. umbellulata var. pectinata

**Figure 3:**
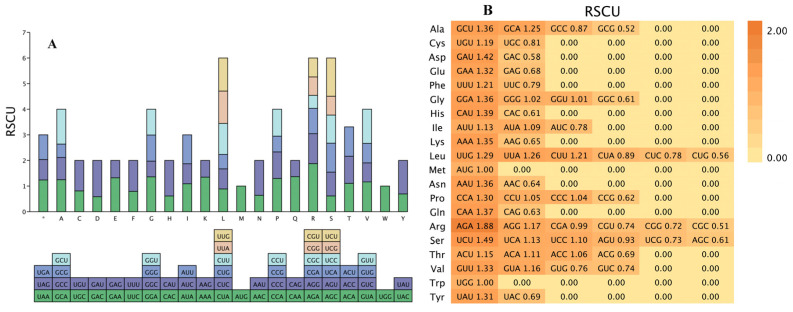
Relative Synonymous Codon Usage (RSCU) analysis of the *S. umbellulata* var. *pectinata* chloroplast genome. (**A**) RSCU heatmap; (**B**) RSCU frequency distribution.

Codon usage bias, measured by the relative frequency of specific codons among synonymous alternatives encoding the same amino acid, quantifies preferential codon selection. When the Relative Synonymous Codon Usage (RSCU) value exceeds 1, the codon is utilized more frequently than expected; an RSCU value equal to 1 indicates no preference. Analysis of the *S. umbellulata* var. *pectinata* chloroplast genome (Figure 3A,B; Table S1) revealed 64 codons encoding 20 amino acids. The AGA codon showed the highest RSCU (1.88), while CGC exhibited the lowest (0.51). Among 32 high-frequency codons (RSCU > 1), 26 (81.25%) terminated with A/U bases, demonstrating a pronounced preference for A/U-ending codons in this chloroplast genome. In the chloroplast genome of *S. umbellulata* var. *pectinata*, there are 117 mononucleotide simple sequence repeats (SSRs), 47 dinucleotide repeats, and only 3 trinucleotide SSRs, while the remaining 4 are tetranucleotide SSRs. The total number of SSRs in the entire genome reaches 171. The results indicate that the discovery of mononucleotide SSRs is crucial for studying the function and structure of the chloroplast genome of *S. umbellulata* var. *pectinata*. Among the detected SSRs, 83.04% consist of A/T, AT/AT, and AAT/ATT as repeat units, which demonstrates a high preference for the usage of bases A and T in the simple repeat sequences of the chloroplast genome of *S. umbellulata* var. *pectinata* (Table 2).

### 3.3. Sequence Analysis of Chloroplast Genome of S. umbellulata var. pectinata and Its Related Taxa

**Figure 4:**
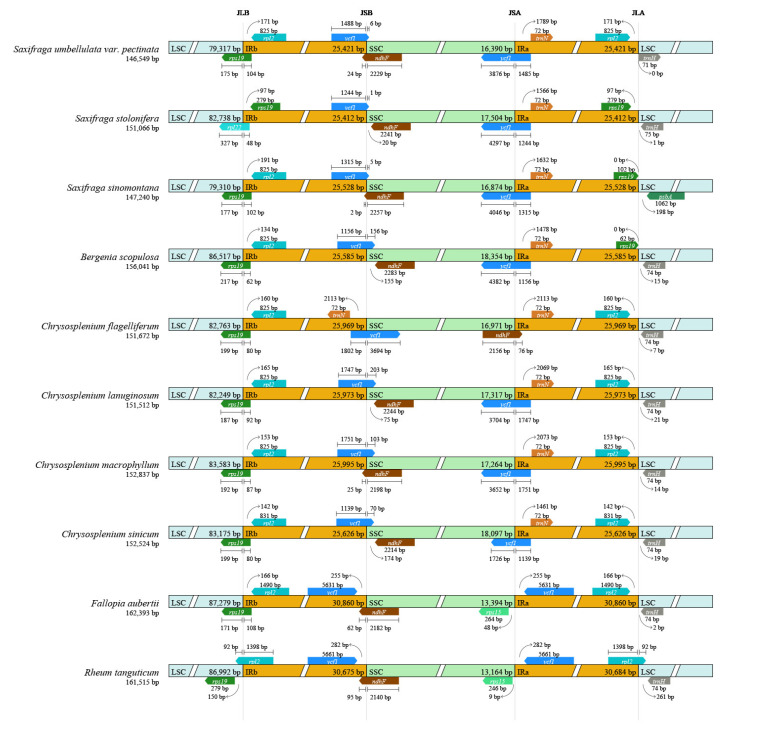

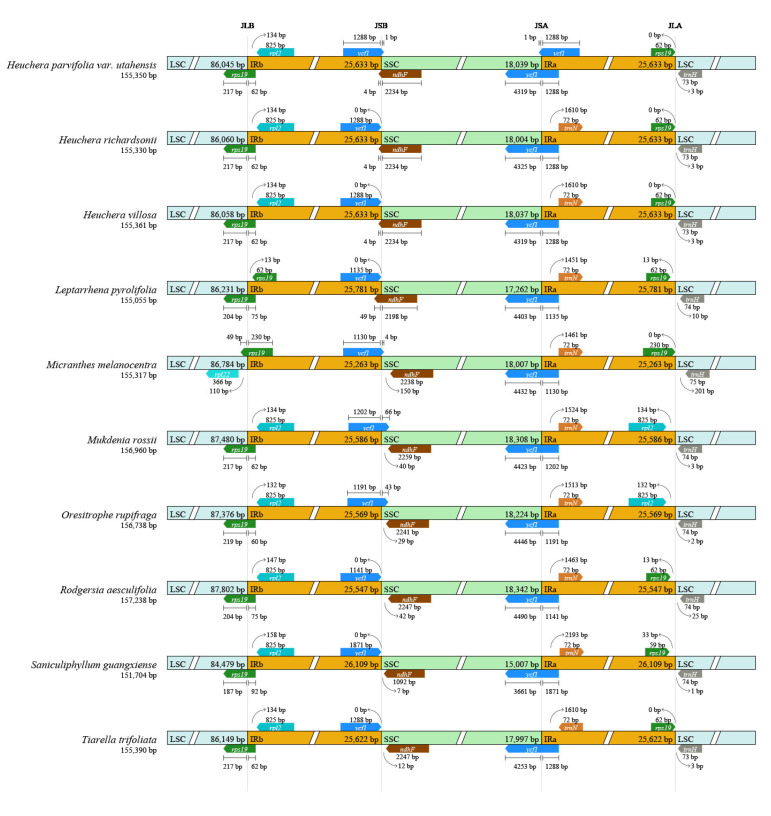
Contraction and expansion of inverted repeat/single-copy (IR/SC) boundary regions in the *S. umbellulata* var. *pectinata* chloroplast genome. Arrows denote the distance between annotated genes and the nearest IR boundary.

At the JLB boundary, with the exception of the *rps19* gene of *S. stolonifera* and *R. tanguticum*, the *rps19* genes of the remaining 18 species are located at the boundary. Specifically, the *rps19* gene of *S. stolonifera* is distributed in the IRb region, while that of *R. tanguticum* is found in the LSC region. Furthermore, apart from *S. stolonifera* and *M. melanocentra*, which possess the *rpl22* gene, all other boundaries contain the *rpl2* gene. Most of the genes are located in the IRb region, at the JSB boundary, where the genes *ndhF* and *ycf1* are distributed. However, only *Chrysosplenium flagelliferum* contains the *trnN* gene. At the JSA boundary, all boundaries except for *Chrysosplenium flagelliferum* exhibit the presence of the *ycf1* gene, while the *trnN* gene is generally found at the JSA boundary. At the JLA boundary, the *trnH* gene is distributed on the LSC side and does not cross the boundary (Figure 4).

**Figure 5:**
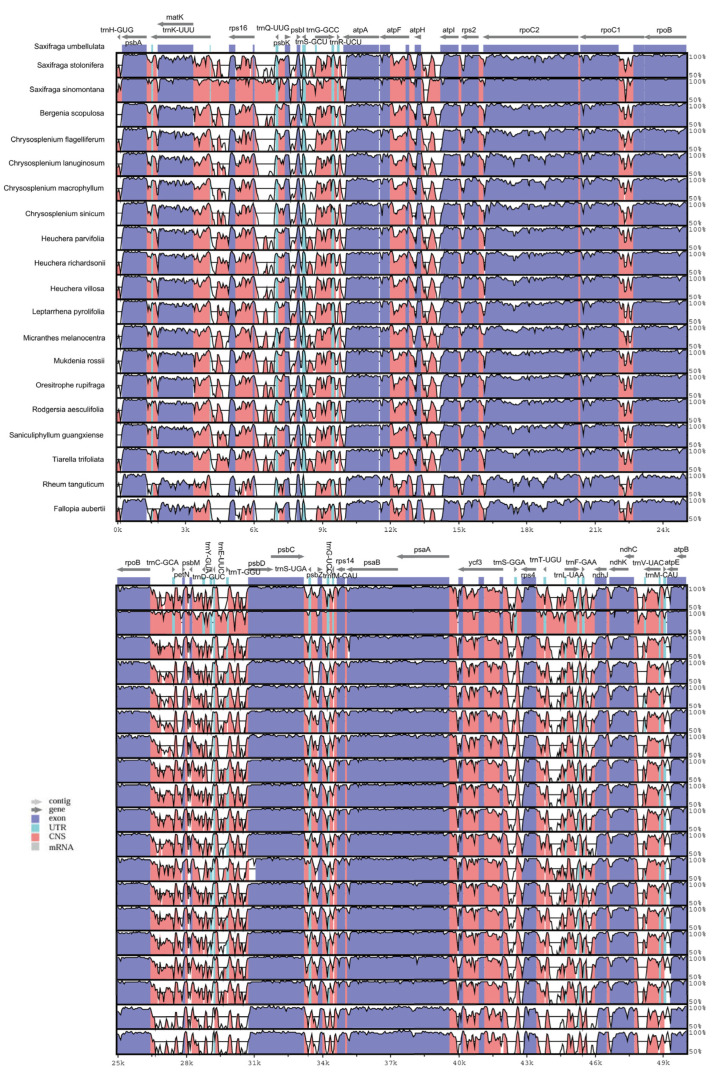

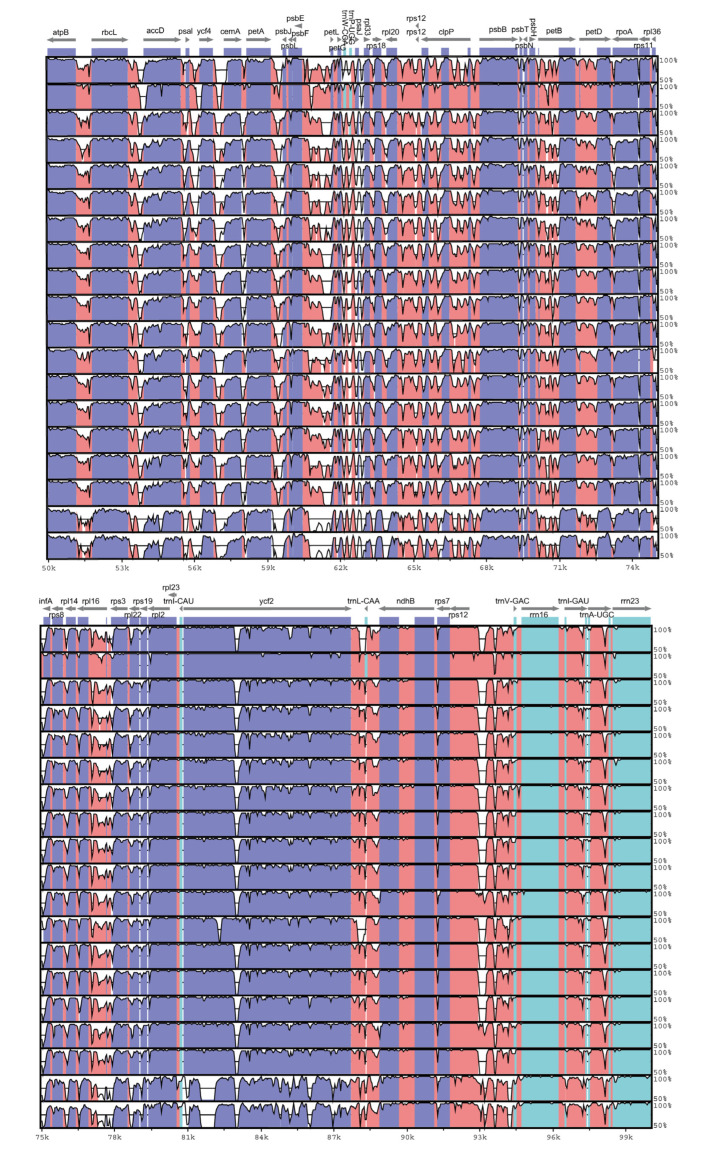

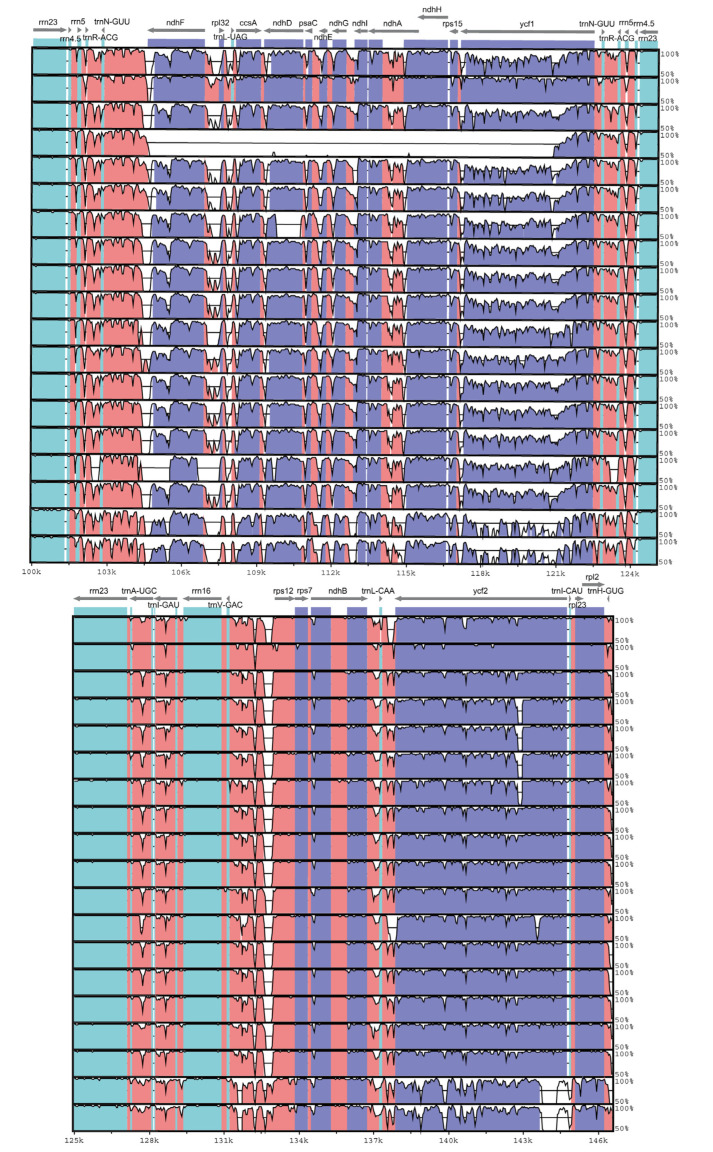
The alignment and comparative analysis of the whole cp genome for twenty *Saxifraga* species. Among them, *S. umbellulata* var. *pectinata* was set as the reference. The horizontal axis represents the coordinates of cp genomes in the alignment result. Exons, introns, and conserved non-coding sequences (CNSs) were marked as different colors.

The chloroplast genomes of *S. umbellulata* var. *pectinata* were further analyzed using the mVISTA method. The analysis revealed that the sequence patterns across the entire chloroplast genome are highly conserved among the studied species. This study included 18 species from the Saxifragaceae family and two outgroups, *R. tanguticum* and *F. aubertii* (as shown in Figure 5). The overall genomic variation is considerable, with non-coding regions exhibiting greater differences than coding regions. Genes with significant variations are concentrated in non-coding regions such as *trnK^UUU^*-*trnR^-UCU^*, *atpH-atpI*, and *rpoB-PsbD*. Additionally, variations are also present in the coding regions of genes such as *rpoC2*, *rpoA*, *ycf2*, *ndhf*, and *ycf1*. Compared to other plants, *B. scopulosa* shows a gene deletion in the region between 103k and 121k. Furthermore, the research results indicate that the protection levels of the *trunk -uuu*, *rpoC2*, *ycf2*, and *rps16* genes in the *F. aubertii* and *R. tanguticum* genomes are relatively low.

### 3.4. Phylogenetic Analysis

**Figure 6:**
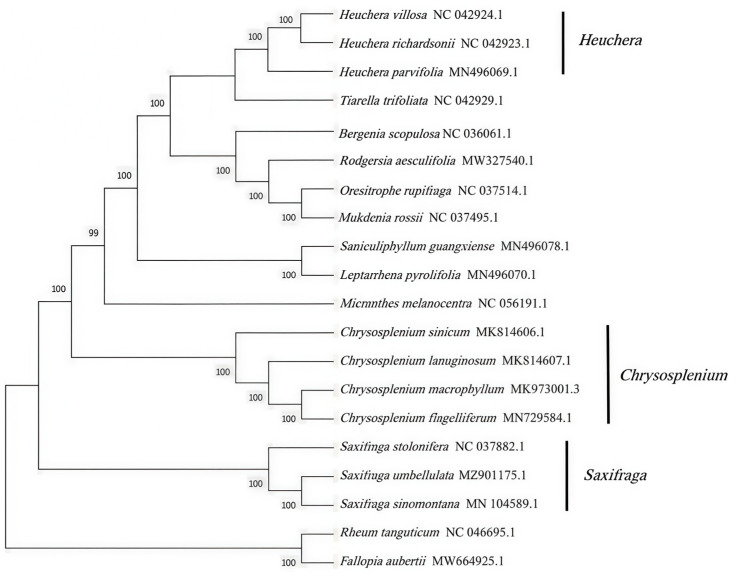
Phylogenetic tree of 20 species based on chloroplast genome.

This study employed neighbor-joining (NJ) analysis in MEGA 7.0 software using chloroplast genome data to infer the phylogenetic relationships among 18 closely related Saxifragaceae species, with *R. tanguticum* and *F. aubertii* serving as outgroups. The bootstrap value—a quantitative measure of branch reliability calculated via resampling methods—was used to assess topological confidence; 1000 bootstrap replicates were completed in this analysis. Results showed that most nodes received 100% bootstrap support, indicating highly reliable phylogenetic reconstruction. Specifically, *S. umbellulata* var. *pectinata* formed a tight cluster with *S. sinomontana* and *S. stolonifera* (100% support), confirming their close affinity (Figure 6), consistent with prior studies [20]. Other genera (e.g., *Chrysosplenium* and *Micranthes*) formed distinct branches with 99–100% support, clearly reflecting genus-level taxonomic boundaries. The outgroups *R. tanguticum* and *F. aubertii* occupied basal positions (100% support), showing significant differentiation from Saxifragaceae species, which validated the appropriateness of outgroup selection.

## 4. Discussion

The chloroplast genome is a cornerstone resource for plant phylogenetics, classification, and molecular evolution. Originating from cyanobacteria [21], it offers a favorable combination: substantial genetic information within a moderately sized molecule (compared to nuclear and mitochondrial genomes), facilitating sequencing. Its relatively slow nucleotide substitution rate and conservative evolution further support robust phylogenetic and species identification studies [22]. Higher plant chloroplast genomes are typically circular, ranging from 108 to 165 kb and containing approximately 80 protein-coding genes [23]. Consistent with this, our sequencing revealed the *S. umbellulata* var. *pectinata* chloroplast genome is 146,549 bp and encodes 131 genes (86 protein-coding). This size aligns with the characteristic range for *Saxifraga* species (146.5–151.1 kb) and falls within the documented variation for sect. *Ciliatae* plastomes (143,479–159,938 bp) [24], demonstrating structural consistency at both genus and section levels.

To resolve evolutionary relationships within Saxifragaceae, we reconstructed a phylogeny using 18 published chloroplast genomes from the family, with *R. tanguticum* and *F. aubertii* as outgroups. The analysis revealed distinct clades: one comprising *Tiarella*, *Heuchera*, *Bergenia*, *Rodgersia*, *Oresitrophe*, *Mukdenia*, *Saniculiphyllum*, and *Leptarrhena*; a separate clade for *Micranthes*; another for *Chrysosplenium*; and a third clade containing the three analyzed *Saxifraga* species. The distant relationship observed between *M. melanocentra* and *Saxifraga* likely reflects its classification within *Micranthes* and/or morphological and environmental divergence. Crucially, *S. umbellulata* var. *pectinata* forms a highly supported clade (100% bootstrap) with *S. sinomontana* and *S. stolonifera*, resolving potential ambiguities in traditional morphology-based classification and providing molecular taxonomic evidence for *Saxifraga*.

We determined the complete chloroplast genome lengths for 18 Saxifragaceae species. All genomes were intact, averaging 154,349 bp in length (range: 146,377 bp in *S. umbellulata* var. *pectinata* to 156,960 bp in *Mukdenia rossii*). While most of the typical 74 angiosperm chloroplast protein-coding genes were retained, evidence of gene transfer, rearrangement, and loss was observed across taxa. The high structural conservation of chloroplast genomes enables comparative identification of mutational hotspots flanked by conserved regions, which serve as valuable markers (e.g., DNA barcodes) for population genetics and phylogenetics. mVISTA analysis confirmed that sequence variation in Saxifragaceae occurs predominantly in non-coding regions, though coding regions also vary. Compared to *S*. *sinomontana* and *S. stolonifera*, *S. umbellulata* var. *pectinata* exhibits loss of the ycf1 gene at the LSC-IRa boundary. In contrast, *S*. *sinomontana* and *S. stolonifera* possess an intact ycf1 gene at this junction. This genomic rearrangement may be associated with its high-altitude adaptation. Additionally, widespread repetitive sequences promote complex structure formation through specific protein binding [25]. SSR analysis of *S. umbellulata* var. *pectinata* revealed single-nucleotide repeats as the most frequent, followed by di-, tri-, tetra-, penta-, and hexanucleotide repeats. Among dinucleotides, AT/TA and TC were predominant, while GC/CG repeats were rare or absent, consistent with the genome’s high AT content. The utilization of AT-biased codons and SSRs rich in A/T may represent adaptive strategies for survival at high altitudes, enhancing translation efficiency under low-temperature stress. These SSRs thus constitute valuable molecular markers for future population studies. Synonymous codon usage analysis showed a preference for A/U-ending codons (RSCU > 1 for 32 codons, 81.25% ending in A/U). Collectively, these findings provide valuable insights for the utilization, development, and conservation of *Saxifraga* resources. The chloroplast genome analysis clarifies the evolutionary position of *S. umbellulata* var. *pectinata* within *Saxifraga*, resolving classification ambiguities and providing a basis for systematic studies of Tibetan Plateau alpine flora. As an endemic Tibetan medicinal plant, functional genes identified within its chloroplast genome may be involved in biosynthetic pathways for secondary metabolites (e.g., flavonoids and alkaloids). The sequence characteristics and expression patterns of these genes offer molecular-level explanations for its traditional medicinal efficacy, particularly anti-inflammatory and antioxidant properties, and lay the groundwork for targeted exploration of active components.

## 5. Conclusions

In recent years, with the rapid advancement of sequencing technologies, an increasing number of researchers have focused on analyzing chloroplast genomes. In this study, we conducted a comparative analysis of the chloroplast genes from 18 species within the genus *Saxifraga*. Our results revealed that the chloroplast genome of *S. umbellulata* var. *pectinata* is a double-stranded circular DNA molecule with a length of 146,549 base pairs (bp) and contains 131 genes in total. These include 86 protein-coding genes, 8 ribosomal RNA (rRNA) genes, and 37 transfer RNA (tRNA) genes. The genome exhibits a preference for codons ending with adenine (A) or uracil (U) bases. Additionally, adenine (A) and thymine (T) are the most abundant types of simple sequence repeats. In terms of phylogenetic analysis, *S. umbellulata* var. *pectinata* shows a close relationship with *S. sinomontana* and *S. stolonifera*, clustering together into a single clade.

## Figures and Tables

**Table 1:** List of genes in the chloroplast genome of *S. umbellulata* var. *pectinata*.

Category	Gene Group	Gene Name
	Subunits of photosystem I	*PsaA*, *psaB*, *psaC*, *psaI*, *psaJ*
	Subunits of photosystem II	*PsbA*, *psbB*, *psbC*, *psbD*, *psbE*, *psbF*, *psbH*, *psbI*, *psbJ*, *psbK*, *psbL*, *psbM*, *psbN*, *psbT*, *psbZ*
	Subunits of NADH dehydrogenase	ndhA*, ndhB*(2), *ndhC*, *ndhD*, *ndhE*, *ndhF*, *ndhG*, *ndhH*, *ndhI*, *ndhJ*, *ndhK*
Photosynthesis	Subunits of cytochrome b/f complex	*petA*, petB*, petD*, *petG*, *petL*, *petN*
	Subunits of ATP synthase	*atpA*, *atpB*, *atpE*, atpF*, *atpH*, *atpI*
	Large subunit of rubisco	*rbcL*
	Subunits photochlorophyllide reductase	*-*
	Proteins of large ribosomal subunit	*rpl14*, rpl16*, *rpl2(2)*, *rpl20*, *rpl22*, *rpl23(2)*, *rpl32*, *rpl33*, *rpl36*
	Proteins of small ribosomal subunit	*rps11*, rps12**(2), *rps14*, *rps15*, rps16*, *rps18*, *rps19*, *rps2*, *rps3*, *rps4*, *rps7(2)*, *rps8*
Expression of related genes	Subunits of RNA polymerase	*rpoA*, *rpoB*, rpoC1*, *rpoC2*
	Ribosomal RNAs	*rrn16(2)*, *rrn23(2)*, *rrn4.5(2)*, *rrn5(2)*
	Transfer RNAs	trnA-UGC*(2), *trnC-GCA*, *trnD-GUC*, *trnE-UUC*, *trnF-GAA*, *trnG-GCC*, trnG-UCC*, *trnH-GUG*, *trnI-CAU(2)*, trnI-GAU*(2), trnK-UUU*, *trnL-CAA(2)*, trnL-UAA*, *trnL-UAG*, *trnM-CAU*, *trnN-GUU(2)*, *trnP-UGG*, *trnQ-UUG*, *trnR-ACG(2)*, *trnR-UCU*, *trnS-GCU*, *trnS-GGA*, *trnS-UGA*, *trnT-GGU*, *trnT-UGU*, *trnV-GAC(2)*, trnV-UAC*, *trnW-CCA*, *trnY-GUA*, *trnfM-CAU*
Other genes	Envelope membrane protein	*cemA*
	Acetyl-CoA carboxylase	*accD*
	c-type cytochrome synthesis gene	*ccsA*
	Translation initiation factor	*infA*
	Maturase	*matK*
	Protease	clpP**
	Conserved hypothetical chloroplast ORF	*ycf1(2)*, *ycf2(2)*, ycf3**, *ycf4*

Notes: Gene*: Gene with one intron; Gene**: Gene with two introns; Gene(2): Number of copies of multi-copy genes.

**Table 2:** SSR in the *Saxifraga umbellulate* var. *pectinata* cp genome.

Repeat Types	Repeat Sequence	Repeat Time														Totality
		3	4	5	6	7	8	9	10	11	12	13	14	15	16	
Mononucleotide	A/T						37	32	17	11	6	4	2	2	1	112
	C/G							1	3	1						5
Dinucleotide	AG/CT		17	1												18
	AT/AT		16	9	4											29
Trinucleotide	AAT/ATT		1	1												2
	ATC/ATG		1													1
Tetranucleotide	AAAG/CTTT	2														2
	AACC/GGTT	1														1
	ACAT/ATGT	1														1

## Data Availability

The original data presented in the study are openly available in GenBank. and the accession numbers are MZ901175.1. (DOI: https://www.ncbi.nlm.nih.gov/nuccore/MZ901175.1, accessed on 1 April 2025).

## Supplementary Materials

The following supporting information can be downloaded at https://www.mdpi.com/article/10.3390/genes16070789/s1, Table S1: SSR information for *Saxifraga umbellulata* var. *pectinata*.
