# Supplementary material for: Comparison of Chloroplast Genome Sequences of Saxifraga umbellulata var. pectinata in Qinghai–Xizang Plateau
Source: Genes (Basel). 2025 Jun 30;16(7):789. doi: 10.3390/genes16070789 (PMC12295303; doi:10.3390/genes16070789)
Supplement: Supplementary file 1 [file genes-16-00789-s001.zip › genes-3719220-supplementary.pdf]

Table S1 SSR information of the chloroplast genome in *Saxifraga umbellulata* var. *pectinata*

AminoAcid	Symbol	Codon	No.	RSCU
*	Ter	UAA	1119	1.2383
*	Ter	UAG	720	0.7968
*	Ter	UGA	872	0.965
A	Ala	GCA	492	1.2471
A	Ala	GCC	342	0.8669
A	Ala	GCG	207	0.5247
A	Ala	GCU	537	1.3612
C	Cys	UGC	458	0.8106
C	Cys	UGU	672	1.1894
D	Asp	GAC	424	0.5848
D	Asp	GAU	1026	1.4152
E	Glu	GAA	1196	1.3223
E	Glu	GAG	613	0.6777
F	Phe	UUC	1336	0.7915
F	Phe	UUU	2040	1.2085
G	Gly	GGA	734	1.3593
G	Gly	GGC	331	0.613
G	Gly	GGG	551	1.0204
G	Gly	GGU	544	1.0074
H	His	CAC	386	0.6127
H	His	CAU	874	1.3873
I	Ile	AUA	1483	1.0899
I	Ile	AUC	1062	0.7805
I	Ile	AUU	1537	1.1296
K	Lys	AAA	1920	1.3474
K	Lys	AAG	930	0.6526
L	Leu	CUA	705	0.8883
L	Leu	CUC	622	0.7837
L	Leu	CUG	446	0.5619
L	Leu	CUU	961	1.2108
L	Leu	UUA	1003	1.2638
L	Leu	UUG	1025	1.2915
M	Met	AUG	813	1
N	Asn	AAC	765	0.6388
N	Asn	AAU	1630	1.3612
P	Pro	CCA	760	1.2953
P	Pro	CCC	609	1.0379
P	Pro	CCG	361	0.6153
P	Pro	CCU	617	1.0516
Q	Gln	CAA	970	1.3672
Q	Gln	CAG	449	0.6328
R	Arg	AGA	964	1.8755
R	Arg	AGG	601	1.1693
R	Arg	CGA	508	0.9883
R	Arg	CGC	261	0.5078
R	Arg	CGG	370	0.7198
R	Arg	CGU	380	0.7393
S	Ser	AGC	465	0.6145
S	Ser	AGU	703	0.9291
S	Ser	UCA	856	1.1313
S	Ser	UCC	830	1.0969
S	Ser	UCG	556	0.7348
S	Ser	UCU	1130	1.4934
T	Thr	ACA	618	1.106
T	Thr	ACC	591	1.0577

T	Thr	ACG	385	0.689
T	Thr	ACU	641	1.1472
V	Val	GUA	648	1.1608
V	Val	GUC	415	0.7434
V	Val	GUG	425	0.7613
V	Val	GUU	745	1.3345
W	Trp	UGG	664	1
Y	Tyr	UAC	676	0.693
Y	Tyr	UAU	1275	1.307
